# Universal Health Coverage for Health Equity: From Principle to Practice; A Response to the Recent Commentaries

**DOI:** 10.34172/ijhpm.2022.7218

**Published:** 2022-03-14

**Authors:** Matthew Fisher, Toby Freeman, Tamara Mackean, Sharon Friel, Fran Baum

**Affiliations:** ^1^Southgate Institute for Health, Society and Equity, Flinders University, Adelaide, SA, Australia.; ^2^College of Medicine and Public Health, Flinders University, Adelaide, SA, Australia.; ^3^The George Institute for Global Health, Sydney, NSW, Australia.; ^4^RegNet School of Regulation and Global Governance, Australian National University, Canberra, ACT, Australia.; ^5^Stretton Health Equity, University of Adelaide, Adelaide, SA, Australia.

 Universal health coverage (UHC) is defined by the World Health Organization (WHO) as when ‘all individuals and communities receive the health services they need without suffering financial hardship’ including ‘health promotion … prevention, treatment, rehabilitation, and palliative care.’^1^ Achieving UHC globally is part of the United Nations’ Sustainable Development Goals and central to the 2018 WHO Global Conference on Primary Health Care Astana Declaration.^2^ Universal systems of primary healthcare (PHC) within countries are widely recognised as central to the task of achieving UHC.^2^ Such systems are crucial to reduce the adverse impacts of both communicable and non-communicable diseases (NCDs) and reduce health inequities within and between countries. However, while the WHO’s and the United Nations’ objectives for UHC seem clear, putting those objectives into practice, with PHC systems capable – over time – of delivering the desired health outcomes, is quite another matter.

 In 2020 we published an article in this journal^3^ examining lessons from the relatively mature Australian system of UHC on how to structure and deliver a system of universal PHC to address NCDs, deliver equity of access to services and, ultimately, promote health equity. (Hereafter we will use ‘equity’ as shorthand for the two latter goals). We identified both strengths and weaknesses of the Australian system in this regard. The journal then sought and published eight commentaries on our article from experts in UHC and PHC from around the globe.^4-11^ Together with our original article, these thoughtful commentaries reinforce our contention that, while commitment to a principle of UHC is welcome, and implies concerns for equity, actual benefits for equity will depend on how UHC is defined and implemented in practice.^6^ In this reply, we describe common themes arising from the commentaries, which highlight key issues for policy makers implementing UHC, and respond to points raised about limitations of our research. Although our research focus (conceived in 2015) was on system responses to NCDs, several commentators noted relevance of the issues raised for the coronavirus disease 2019 (COVID-19) pandemic.

## Learning From Mature Systems

 We proposed that countries seeking to implement UHC to tackle NCDs and advance equity could learn from Australia’s nearly 40 years of experience.^3^ In general, the commentaries affirmed this proposition, examining the concordance of the issues we raised with similar concerns globally and in other jurisdictions, including low- and middle-income countries^7^ and countries beginning to implement UHC systems. Among other things, mature systems show that early choices about system design are crucial, because they will do much to determine long-term performance and, once in place, are difficult to change.^6,11^

## Defining Universal Health Coverage

 International debate on UHC has focused on systems of universal financial coverage for the costs of using healthcare. Several commentators argued that choices about public or private health insurance systems^6^ and other funding mechanisms^11^ will have significant effects on equity. However, most also argued that, to optimise benefits, policy makers must go beyond financial coverage and shift to a more holistic and rights-based view of UHC^10^ encompassing *inter alia* PHC systems, models of care, public health regulation and action on social determinants of health.^5-7,9,10^

## Conceptions of Health and Healthcare

 Several commentators concurred with our view that narrow biomedical and behaviourist views of health constrain the role of PHC systems to prevent and manage NCDs: favouring funding structures and/or models of care limited to episodic primary medical care and individualised ‘lifestyle’ behaviour change and obstructing the multi-sectoral, comprehensive, community-engaged approaches required to manage and prevent NCDs and promote population health effectively and equitably,^3,8^ including work with other sectors to address social determinants of health.^4,6,7,9^

## Role of the Private Sector

 As we found,^3^ design and implementation of UHC systems by governments can involve or intersect with the corporate interests of large private companies or small businesses in a variety of ways. Commentaries broadly concurred with our findings that governments implementing UHC should be wary of the role of private sector organisations and interests, having the potential to produce *inter alia*: inequities in availability or affordability of PHC services; reinforcement of limited biomedical approaches to care^7^; and political constraints on public health regulation of commercial determinants of health.^5,8^ Several examples of inequities arising in health systems combining public and private funding or delivery structures were noted.^5,7^ Loewenson^6^ highlights the role of neoliberal politics in increasing these risks; Woodruff^11^ emphasises the role of funding structures as policy mechanisms to control them. Paremoer^8^ calls for more public engagement in policy making to counter corporate influence. Nandi^7^ contrasts the benefits of recent policies to strengthen publicly funded PHC in India, including community health workers, with inequitable effects of a privatised and commercialised hospital sector.

## Universality, Targeting and Devolution

 Universal financial coverage is certainly likely to be better for equity than any PHC system where access depends on private ability to purchase private insurance or pay for services directly. However, as several commentators discussed, ostensibly universal systems can still limit equity of access in practice, for example, by limiting coverage to selected services, failing to control out of pocket expenses for service users, or failing to match the distribution of resources or models of care to the needs of different population groups.^4,6,9^ Thus, in practice, universal PHC systems aiming for equity are likely to require funding structures both for equitable distribution across population groups and targeting to meet specific needs. Woodruff^11^ endorses our point about the potential for devolved funding structures, placing more decision-making power in localised organisations, to tailor services to local needs; an issue we explore further in a more recent article in this journal.^12^

## COVID-19

 Although our focus was on PHC systems’ ability to address rising rates of NCDs, several commentators considered implications and possible lessons for health system responses to the COVID-19 pandemic. In general terms two arguments were made: that many of the issues raised from our research are equally applicable to achieving PHC systems to manage and prevent communicable diseases^6^; and that the pandemic itself may provide some impetus for reforms in the directions we have discussed^4,9^ especially because it has brought health inequities to the fore.^13^

## Limitations of Our Work

 Berg^4^ and Paremoer^8^ say our research failed to explore service delivery issues such as practitioner discrimination, which undermine equity of access and acceptability of services for minority groups and require improved health professional training. We agree we did not delve into these important issues but argue that this does not invalidate our findings or conclusions. Paremoer^8^ says we failed to consider how ‘justified scepticism of public healthcare might generate political support for exactly the kind of inequitable UHC financing and service provision models this paper warns against.’ This is indeed not an issue we considered but, on the face of it, we would agree that in the current political climate public dissatisfaction with public healthcare systems could indeed be manipulated in this way.

 Overall, the commentaries reinforce the need for health systems that go beyond UHC to those that are grounded in comprehensive PHC with all the attributes from the still highly relevant WHO Alma Ata Declaration.^14^

## Acknowledgements

 We would like to acknowledge and thank our colleagues from the Centre of Research Excellence on the Social Determinants of Health Equity.

## Ethical issues

 Not applicable.

## Competing interests

 MF reports grants from National Health and Medical Research Council, during the conduct of the study.

## Authors’ contributions

 MF read commentaries and drafted the paper. TF read commentaries and reviewed draft versions of the paper. TM and SF reviewed draft versions of the paper. FB read commentaries and reviewed draft versions of the paper.

## Funding

 Preparation of this article was conducted under the Centre of Research Excellence on the Social Determinants of Health Equity, funded by the Australian National Health and Medical Research Council (GNT1078046). The funder had no role in: study design; collection, analysis and interpretation of data; writing of the article; or the decision to submit it for publication.
